# Transition readiness among Chinese adolescents with chronic diseases: the promoting role of self-efficacy and willingness to engage in healthcare

**DOI:** 10.1186/s12913-025-13509-8

**Published:** 2025-11-21

**Authors:** Xiao Wu, Yun Jiang, Ying Luo, Shi Huang, Xiuli Qin, Yonghong Yi, Huiyun Zhu, Junli Yi, Zhenyun Zhu, Genzhen Yu

**Affiliations:** Tongji Hospital, Tongji Medical College, Huazhong University of Science and Technology, Wuhan, China

**Keywords:** Adolescent, Chronic disease, Transition readiness, Self-efficacy, Willingness to engage in health care

## Abstract

**Background:**

In China, research on transition readiness among adolescents with chronic diseases remains exploratory. Cultural and healthcare system differences limit the applicability of Western findings. This study investigates the effects of self-efficacy and willingness to engage in healthcare on transition readiness, aiming to develop culturally informed interventions for the Chinese context.

**Methods:**

A cross-sectional study recruited 205 adolescents from a tertiary hospital in Wuhan through convenience sampling from March to October 2023. The study used validated scales to evaluate the following indicators: transition readiness (using The Self-Management and Transition to Adulthood with Rx = Treatment (STARx) Questionnaire), self-efficacy (through the General Self-Efficacy Scale), and healthcare engagement (using the questionnaire of willingness to engage in healthcare). Data analysis was conducted using SPSS 25.0 software and processed through methods such as independent sample t-test, one-way analysis of variance, pearson correlation analysis and multiple linear regression.

**Results:**

The overall level of transition readiness among adolescents with chronic diseases was moderate (mean score = 3.19 ± 0.58). Adolescents aged 13–15 years (β = 0.210, *P* = 0.003) and those over 15 years (β = 0.376, *P* < 0.001), along with self-efficacy (β = 0.229, *P* = 0.001) and willingness to engage in healthcare (β = 0.234, *P* < 0.001), were identified as independent positive predictors of transition readiness among adolescents with chronic diseases. Conversely, hemophilia (β= -0.350, *P* = 0.001), recurrent disease (β= -0.120, *P* = 0.037), disease deterioration (β = -0.155, *P* = 0.009), and maternal caregiving (β = -2.574, *P* = 0.011) emerged as significant negative predictors of transition readiness.

**Conclusions:**

This study reveals the key factors affecting the transition readiness of adolescents with chronic diseases, and emphasizes that a multidimensional and sustainable transition support system should be constructed from psychological empowerment, family involvement and individual differences to provide a basis for improving their long-term health management ability and quality of medical care interface.

## Background

With advancements in medical technology, diagnostics, and treatment, the prevalence of chronic diseases among children has increased, while mortality rates have significantly declined [1]. Currently, more than 10% of adolescents are living with chronic conditions, and approximately 750,000 pediatric patients transition from pediatric to adult healthcare services each year [2].

Health care transition refers to the process of shifting adolescents and young adults with chronic conditions from a pediatric, parent-supervised care model to an independent, patient-centered adult care model [3, 4]. Transition readiness is a key quantitative indicator of health care transition, reflecting the adolescent’s overall capacity to manage this shift [4, 5]. Higher levels of transition readiness are associated with reduced risks of treatment interruption, non-adherence, and disease exacerbation, and are critical for a successful transition [5, 6].

However, only about 40% of patients successfully complete the transition process [7], with many experiencing unmet healthcare needs and insufficient transitional care support [8].

Although this field has been extensively studied internationally, cultural and healthcare system differences limit the applicability of these findings in China. On one hand, the family-centered care model often places parents at the center of medical decision-making, restricting adolescents’ autonomy and engagement [9]. On the other hand, while over 96% of pediatric care in China is delivered by general hospitals, most have yet to establish standardized transition management mechanism [10], leaving adolescents with chronic diseases facing significant challenges during the shift from pediatric to adult care [11].

Self-efficacy refers to an individual’s perceived capability to achieve specific behavioral goals within a given domain [12]. Willingness to engage in healthcare reflects a patient’s willingness to engage in medical care and rehabilitation through strategies such as shared decision-making, care involvement, and advocacy [9]. Previous studies have shown that both self-efficacy and healthcare engagement play critical roles in promoting self-management among adolescents with chronic conditions [9, 13]. However, few studies have examined their relationship with transition readiness, and the strength of these associations remains unclear.

In recent years, China has begun to pay greater attention to transition readiness among adolescents with chronic conditions. However, research on its influencing factors remains in the exploratory stage [14]. This study aims to investigate the impact of two key factors—general self-efficacy and willingness to engage in healthcare on transition readiness, with the goal of providing a theoretical foundation for developing culturally appropriate transition interventions for Chinese adolescents with chronic diseases.

## Methods

### Study design and setting

This descriptive cross-sectional study was conducted at a major tertiary teaching hospital in Wuhan, China, between March and October 2023.

### Participants

Convenience sampling method was used to select the study population for this study. Inclusion criteria were (1) aged 12–18 years, (2) hospitalized patients, (3) the disease met the characteristics of chronic diseases in children [15], and diagnosed with diabetes, chronic kidney disease, juvenile idiopathic arthritis, epilepsy, hemophilia, or inflammatory bowel disease;, and (4) voluntary participation by the person himself or herself and his or her guardian. The exclusion criteria were (1) suffering from psychological diseases or mental disorders (2) critical illness. The sample size was roughly calculated based on the needs of the multiple linear regression model, in which the sample size should be more than 5 to 10 times the number of independent variables [16]. The number of independent variables in this study was 24, and taking into account the 20% invalid questionnaires, the required sample size was 144 to 288. 210 questionnaires were distributed and those with missing demographic data were excluded. Finally, 205 study participants were included in this study.

## Measurements

### Sociodemographic characteristics questionnaire

The sociodemographic characteristics questionnaire was designed by the researchers. The questionnaire contains 12 items covering general information about the child, disease-related information, and family information.

### The self-management and transition to adulthood with Rx = Treatment (STARx) questionnaire

The STARx questionnaire was originally developed by Ferris et al. [17] to measure the readiness of chronically ill adolescent patients during the transition from pediatrics to adulthood. Huang et al. sinicized and cross-culturally adapted the scale and validated its application to a group of chronically ill adolescents in a Chinese general hospital [4]. The results showed that the content validity of the scale was 0.92 and the Cronbach’s alpha coefficient was 0.83, indicating that the scale has good reliability and validity. The Chinese version of the scale consists of 18 items covering 6 subscales: medication management, provider communication, engagement during appointments, disease knowledge, adult health responsibilities and resource utilization. The scale is rated on a 5-point Likert scale (1 = “never” to 5 = “always”), with higher scores indicating higher transition readiness. In this study, the Cronbach’s alpha coefficient for the scale was 0.93.

### The general self-efficacy scale

Self-efficacy was assessed by the General Self-Efficacy Scale, which was revised by Chinese scholar Wang Caikang et al. [18]. The scale consists of 10 items, which are rated on a 4-point Likert scale (1 = “not at all correct”, 4 = “completely correct”), with a total score of 10–40, with higher scores indicating higher self-efficacy. The original Cronbach’s alpha of the scale was 0.87, and the half reliability was 0.90. In this study, the Cronbach’s alpha was 0.92.

### The questionnaire of willingness to engage in healthcare

In this study, we used the Health Care Participation Intention Questionnaire developed by Wu Yaping et al. [19] and revised by Wu Qing et al. [20]. The scale was used to assess the health care participation willingness of Chinese hospitalized patients. After being adapted for chronically ill adolescents by Chen Wenjin et al. [9], the scale retained the original 33 entries and six dimensions: participation in curative care, participation in information interaction, participation in medical decision-making, participation in diagnosis and treatment decision-making, participation in claims, and participation in questioning and monitoring. The scale was scored on a 5-point Likert scale (1 = very reluctant, 5 = very willing). The total score ranged from 33 to 165, with higher scores indicating higher willingness to engage in health care. In this study, the mean score of the entries was set at < 3 as a lower level, 3 to 4 as a medium level, and >4 as a higher level. The Cronbach’s alpha coefficient for the scale in this study was 0.868.

### Data collection

In this study, data were collected through questionnaire method. All adolescents and their parents were made aware of the details of the purpose and procedures of the study and signed a written informed consent form prior to the commencement of the survey. Data collection was conducted from March to October 2023 through the Questionnaire Star platform (http://www.wjx.cn) by uniformly trained enumerators. Participants accessed and completed the questionnaire online by scanning a QR code. To avoid duplicate submissions, the system automatically recorded device IDs and limited submissions to one per device. The questionnaire took approximately 20 min to complete; responses submitted within 15 min were considered invalid and excluded. No personally identifiable information was collected and all data were used for research purposes only.

### Statistical analysis

All statistical analyses were performed using IBM SPSS Statistics, Version 25.0. Quantitative data were presented as mean ± standard deviation. Comparisons between groups were analyzed using independent samples t-tests and one-way analysis of variance (ANOVA). Qualitative data are described using frequencies and percentages. Pearson correlation analysis was used to examine the relationships among general self-efficacy, willingness to engage in healthcare, and transition readiness. Multiple linear regression analysis was employed to identify factors influencing transition readiness in adolescents with chronic conditions. Statistical significance was set at *P* < 0.05.

## Results

### Sociodemographic data

As shown in Table 1 and 205 participants were predominantly male (73.7%) and 53.2% were concentrated in the age group of 13–15 years. Family characteristics showed 72.7% were non-only children and 67.3% were from nuclear families. Disease distribution had the highest proportion of renal disease (44.4%), 41.0% had a disease duration of ≤ 5 years, 87.7% had no comorbidities, and 76.6% were stable. Regarding caregiver characteristics, 77.1% of the primary caregivers were mothers, and more than half (62.9%) of the caregivers only had a junior high school education.

### Scores on transition readiness, self-efficacy and willingness to engage in healthcare

Adolescents with chronic diseases demonstrated moderate overall transition readiness (mean = 3.19 ± 0.58; range 1–5). Disease knowledge scored highest (3.68 ± 0.83), indicating good condition understanding. However, engagement during appointment (2.85 ± 0.97) and resource utilization (2.78 ± 0.89) were significantly lower, suggesting challenges in proactive treatment involvement and resource use. Self-efficacy was slightly above the midpoint (mean = 2.53 ± 0.55; range 1–4), indicating room for improvement. Overall healthcare engagement willingness was moderate (mean = 3.80 ± 0.71; range 1–5). Willingness for participation in curative care was highest (3.96 ± 0.76), demonstrating cooperation readiness, while participation in medical decision-making scored lowest (2.27 ± 0.51), underscoring a substantial engagement gap in key health decisions (See Table 2 for details).

### Univariate analysis of transitional readiness

The univariate analysis in Table 1 revealed significant differences in transition readiness scores across demographic and clinical characteristics. Female participants demonstrated significantly higher readiness scores (61.09 ± 9.09) compared to males (56.26 ± 10.68). A positive age-dependent trend was observed, with the lowest scores in the ≤ 12 years group (52.50 ± 12.31) and the highest in the > 15 years group (61.20 ± 8.44). Only children showed significantly better preparedness (60.68 ± 11.65) than those with siblings (56.36 ± 9.79). Significant variations emerged across chronic disease types, where patients with chronic kidney disease exhibited the highest readiness (60.91 ± 10.10), while those with inflammatory bowel disease scored lowest (51.00 ± 9.11). Clinically stable patients achieved the highest scores (58.61 ± 10.74), whereas patients with recurrent conditions scored lowest (53.88 ± 8.54). Participants requiring > 2 daily medications showed superior transition readiness (59.80 ± 10.21). Notably, patients with paternal caregivers scored significantly higher (63.19 ± 13.61) than those with maternal caregivers (56.51 ± 9.96). All reported differences were statistically significant (*P* < 0.05).

### Correlation analysis of transition readiness, self-efficacy and willingness to engage in healthcare

As shown in Table 3, correlation analysis revealed that transition readiness was significantly but weakly positively correlated with self-efficacy (*r* = 0.174, *P* < 0.05). In contrast, a moderate positive correlation was observed between transition readiness and the total score of willingness to engage in healthcare (*r* = 0.409, *P* < 0.01), with significant positive associations across all subdomains (*P* < 0.01). Among these subdomains, participation in medical decision-making exhibited the strongest correlation with transition readiness (*r* = 0.405, *P* < 0.01), whereas questioning and supervision showed the weakest association (*r* = 0.322, *P* < 0.01). Overall, willingness to engage in healthcare(particularly in medical decision-making) demonstrated a more robust relationship with transition readiness compared to other factors.

### Linear multivariate regression analysis (Enter)

A multiple linear regression model was constructed with the total transition readiness score as the dependent variable, incorporating variables that showed significance in univariate analysis along with core variables (self-efficacy and willingness to engage in healthcare). The independent variables were coded as follows: gender: male = 0, female = 1; age: ≤12 years (0,0), 13–15 years (1,0), > 15 years (0,1); disease type: kidney disease (reference, 0,0,0,0), hemophilia (1,0,0,0), juvenile idiopathic arthritis (0,1,0,0), epilepsy (0,0,1,0), inflammatory bowel disease (0,0,0,1); disease status: stable (0,0), recurrent (1,0), progressive (0,1); daily medications: ≤2 = 0, > 2 = 1; primary caregiver: father (reference, 0,0,0), mother (1,0,0), grandparents (0,1,0), others (0,0,1).

The multivariate linear regression model (Table 4) explained 38.6% of the total variance in transition readiness. Results identified age, self-efficacy, and willingness to engage in healthcare as positive predictors. For every one-unit increase in the willingness to engage in healthcare, transition readiness increased by 0.104 points (B = 0.104, 95% CI: 0.048–0.160). Similarly, a one-unit increase in self-efficacy was associated with a 0.436-point increase in transition readiness (B = 0.436, 95% CI: 0.189–0.682). Age demonstrated a significant gradient effect: the > 15 years group showed the highest readiness, scoring 9.42 points higher than the ≤ 12 years group (B = 9.42, 95% CI: 5.881–12.958); the 13–15 years group scored 4.401 points higher than the ≤ 12 years group (B = 4.401, 95% CI: 1.486–7.351). Negative predictors included hemophilia, disease progression, and maternal caregiving. Specifically: among disease types, hemophilia patients scored lowest, with 7.941 points lower than kidney disease patients (B=-7.941, 95% CI: -12.510 to -3.371). For disease status, progressive cases showed the greatest decline (8.916 points lower than stable cases, B=-8.916, 95% CI: -15.565 to -0.187), while recurrent cases scored 3.135 points lower (B=-3.135, 95% CI: -6.082 to -0.188). Maternal caregivers were associated with 5.133 points lower readiness than paternal caregivers (B=-5.133, 95% CI: -9.066 to -1.199). After adjusting for confounders, willingness to engage in healthcare, self-efficacy, age, hemophilia, disease status, and maternal caregiving remained independently significant predictors of transition readiness.

**Table 1:** The general demographic data and the univariate analysis of the adolescent with chronic illness

Variables		*n*(%)	STARx score(${\rm{\bar X}}$±s)	Text value	*P* value
Gender	Male	151(73.7)	56.26 ± 10.68	-2.959	**0.003**
	Female	54(26.3)	61.09 ± 9.09		
Age	≤ 12 years old	50(24.4)	52.50 ± 12.31	9.613	**0.000**
	13 ~ 15 years old	109(53.2)	58.30 ± 9.55		
	>15 years old	46(22.4)	61.20 ± 8.44		
Whether an only child	Yes	56(27.3)	60.68 ± 11.65	2.670	**0.008**
	No	149(72.7)	56.36 ± 9.79		
Disease diagnosis	Chronic kidney disease	91(44.4)	60.91 ± 10.10	8.669	**0.000**
	Hemophilia	63(30.7)	52.67 ± 9.03		
	Juvenile idiopathic arthritis	28(13.7)	60.36 ± 8.55		
	Epilepsy	10(4.9)	58.10 ± 14.55		
	Inflammatory bowel disease	13(6.3)	51.00 ± 9.11		
Duration of illness	≤ 5 years	84(41)	59.36 ± 10.51	2.991	0.052
	5 ~ 10 years	40(19.5)	57.98 ± 9.36		
	>10 years	81(39.5)	55.43 ± 10.72		
Comorbidity with other chronic diseases	Yes	25(12.2)	58.80 ± 10.17	0.412	0.521
	No	180(87.7)	57.36 ± 10.48		
Disease status	Stable condition	157(76.6)	58.61 ± 10.74	3.643	**0.028**
	Recurrent condition	41(20)	53.88 ± 8.54		
	Exacerbation	7(3.4)	54.86 ± 10.88		
The kinds of Daily oral medication	≤ 2	103(50.2)	55.29 ± 10.31	9.912	**0.002**
	>2	102(49.8)	59.80 ± 10.21		
Primary caregiver	Father	21(10.2)	63.19 ± 13.61	3.305	**0.021**
	Mother	158(77.1)	56.51 ± 9.96		
	Grandparents	17(8.3)	57.47 ± 9.23		
	Other	9(4.4)	62.44 ± 9.41		
Family structure	The core family	138(67.3)	56.73 ± 10.28	1.355	0.251
	The stem family	28(13.7)	58.79 ± 9.50		
	Single-parent family	8(3.9)	63.75 ± 12.26		
	The incomplete family	22(10.7)	11.95 ± 2.55		
	The atavism family	9(4.4)	10.33 ± 3.44		
Caregiver’s education level	Primary school and below	38(18.5)	55.58 ± 10.73	0.960	0.413

Bold font is used in the table to highlight *P*-values that are less than 0.05, indicating a statistically significant difference

**Table 2:** The scores of each scale among adolescents with chronic diseases

Variables	The total score (${\rm{\bar X}}$±s)	The entry average score (${\rm{\bar X}}$±s)
**The STARx**	57.54 ± 10.48	3.19 ± 0.58
Medication communication	12.74 ± 2.56	3.19 ± 0.64
Provider communication	10.14 ± 2.61	3.38 ± 0.87
Engagement during appointment	8.54 ± 2.91	2.85 ± 0.97
Disease knowledge	11.03 ± 2.48	3.68 ± 0.83
Adult health responsibilities	6.77 ± 1.72	3.38 ± 0.86
Resource utilization	8.33 ± 2.65	2.78 ± 0.89
**The self-efficacy**	25.29 ± 5.52	2.53 ± 0.55
**Willingness to engage in healthcare**	125.42 ± 23.57	3.80 ± 0.71
Participation in curative care	39.57 ± 7.61	3.96 ± 0.76
Participation in information interaction	34.21 ± 6.84	3.80 ± 0.76
Participation in medical decision -making	11.33 ± 2.53	2.27 ± 0.51
Participation in diagnostic and medical decision -making	18.31 ± 3.88	3.66 ± 0.78
Participation in claims	11.46 ± 2.31	3.82 ± 0.77
Participation in questioning and monitoring	10.54 ± 2.29	3.51 ± 0.76

**Table 3:** Bivariate correlations between self-efficacy, the intention to participate in health care and transition readiness

Variables	The transition readiness	Medication communic-ation	Provider communica- tion	Engagement during appointment	Disease knowle- dge	Adult health responsibiliti-es	Resource utilization
The self-efficacy	0.174*	-0.205**	0.284**	0.088	0.254**	0.209**	0.137
Willingness to engage in healthcare	0.409**	0.176*	0.286**	0.280**	0.364**	0.198**	0.390**
Participation in curative care	0.379**	0.220**	0.243**	0.236**	0.319**	0.203**	0.359**
Participation in information interaction	0.388**	0.157*	0.253**	0.306**	0.346**	0.154*	0.374**
Participation in medical decision -making	0.359**	0.146*	0.237**	0.253**	0.357**	0.170*	0.323**
Participation in diagnosis and treatment decision-making	0.405**	0.114	0.339**	0.286**	0.340**	0.195**	0.400**
Participation in claims	0.385**	0.154*	0.280**	0.260**	0.346**	0.179*	0.370**
Participation in questioning and monitoring	0.322**	0.100	0.263**	0.161*	0.334**	0.207**	0.297**

**: *P*<0.01, * *P*<0.05

**Table 4:** Linear regression on overall STARx score (*n* = 205)

Variable	The STARx score			*t*	*P* value	*95*%*CI*	
	B	SE	β			Lower	Upper
	36.387	5.207		6.988	**0.000**	26.115	46.658
Female	2.560	1.468	0.108	1.744	0.083	-0.336	5.457
13 ~ 15 years old	4.401	1.477	0.210	2.979	**0.003**	1.486	7.315
>15 years old	9.420	1.794	0.376	5.251	**0.000**	5.881	12.958
An only child	2.488	1.340	0.106	1.858	0.065	-0.154	5.131
Hemophilia	-7.941	2.316	-0.350	-3.428	**0.001**	-12.510	-3.371
Juvenile idiopathic arthritis	-0.091	1.906	-0.003	-0.048	0.962	-3.851	3.668
Epilepsy	-0.191	3.384	-0.004	-0.056	0.955	-6.866	6.485
Inflammatory bowel disease	-4.693	2.936	-0.109	-1.599	0.112	-10.484	1.098
Recurrent condition	-3.135	1.494	-0.120	-2.098	**0.037**	-6.082	-0.187
Exacerbation	-8.916	3.370	-0.155	-2.646	**0.009**	-15.565	-2.268
>2 kinds	-2.146	1.938	-0.103	-1.108	0.269	-5.969	1.676
Mother	-5.133	1.994	-0.206	-2.574	**0.011**	-9.066	-1.199
Grandparents	-0.092	2.803	-0.002	-0.033	0.974	-5.621	5.438
others	0.411	3.402	0.008	0.121	0.904	-6.300	7.122
Self-efficacy	0.436	0.125	0.229	3.485	**0.001**	0.189	0.682
Willingness to engage in healthcare	0.104	0.028	0.234	3.665	**0.000**	0.048	0.160

B: Unstandardized Beta, SE: standard error, β: standardized Beta, CI: confidence intervals

R^2^ = 0.434, Adjusted R^2^ = 0.386

## Discussion

This study is the first in China to validate the synergistic role of healthcare engagement willingness and self-efficacy in promoting transition readiness among adolescents with chronic conditions. Using cross-sectional data, we identified key modifiable factors influencing transition readiness, providing empirical evidence to support the development of localized transition support programs.

In this study, transition readiness among adolescents with chronic conditions was at a moderate level. The highest mean score was observed in disease knowledge, while resource utilization and engagement in care had the lowest scores. This “high knowledge–low behavior” pattern aligns with findings by Tornivuori et al. [21], suggesting that while adolescents possess theoretical understanding of their condition, they often lack the ability to translate knowledge into autonomous actions. This highlights the need for healthcare providers to incorporate behavior-oriented education—such as scenario-based simulations—into disease education programs [22].

This study found that adolescents with chronic conditions demonstrated generally low willingness to engage in healthcare, particularly in medical decision-making. These findings are consistent with the results reported by Chen Wenjin [9] for adolescent populations but significantly lower than those observed in adult groups [23, 24]. This disparity may be attributed to traditional family structures, cultural norms, and adolescents’ limited medical knowledge [25]. While total willingness to engage in healthcare was a positive predictor of transition readiness, regression analysis identified participation in medical decision-making as the strongest predictor, aligning closely with Sattoe et al.’s advocacy for patient empowerment and active involvement [26]. However, the notably low willingness to engage in decision-making in this study highlights an urgent need to enhance decision-making training for adolescents with chronic illnesses [27]. Healthcare providers are encouraged to implement shared decision-making exercises and develop pediatric decision aids in collaboration with families. A gradual empowerment approach—shifting from parent-led to adolescent-led care with parental support—may help adolescents assume increasing responsibility for their health management and improve their transition readiness [9].

This study found that general self-efficacy among adolescents with chronic conditions was at a moderately low level, consistent with previous research [28]. Although self-efficacy showed only a weak correlation with transition readiness, it emerged as a significant predictor in the regression model. This suggests that, after controlling for demographic factors, self-efficacy may indirectly enhance readiness by strengthening confidence in disease management [29]. Despite often having a long disease history, adolescents in puberty may lack confidence and motivation due to emotional fluctuations, heightened self-awareness, and resistance to disease management [30]. As a key determinant of health behavior, self-efficacy contributes to an individual’s sense of control and ability to self-manage chronic conditions [31]. Existing intervention studies have confirmed its positive impact in adult populations [32, 33]. These findings highlight the need for healthcare providers to focus on fostering self-efficacy during the transition period. Strategies may include virtual skill-building programs [34] and the establishment of peer role model networks [35], aiming to strengthen adolescents’ confidence and capacity for independent health management.

This study observed an age-related improvement in transition readiness among adolescents with chronic conditions. The 13–15 years group demonstrated 4.401 points higher readiness than the ≤ 12 years group, with a more pronounced increase (>15 years group) consistent with established developmental trajectories of transition readiness [21, 36]. This progression likely reflects cognitive maturation, developing autonomy, and heightened health responsibility. Clinical guidelines recommend systematic transition interventions starting at age 12 [37], establishing foundational skills through early engagement. Healthcare providers and parents should therefore prioritize transition needs during this critical developmental window. Based on this finding, it is recommended that medical staff implement a stratified and progressive intervention strategy: for the group aged ≤ 12, use gamification design to stimulate interest and initially cultivate their awareness of health care and willingness to engage; for adolescents aged 13–15, strengthen micro-decision-making training to enhance their decision-making confidence and skills; for patients over 15, through role-playing, independent record-keeping and other practical methods, strengthen their doctor-patient communication and self-management abilities to facilitate a smooth transition to adult medical models.

This study found significant differences in the transition readiness among adolescents with different chronic diseases. The average transition readiness of hemophilia patients was 7.941 points lower than that of nephropathy patients. Existing research [38] indicates that a longer disease duration may provide more opportunities to develop disease knowledge and skills, which is conducive to enhancing transition readiness. However, this study found that children with hemophilia, who had the longest average disease duration, had the lowest transition readiness. This might be related to their lack of awareness of the serious consequences of improper disease management [39]. This suggests that compared to the mere length of the disease course, disease characteristics and their impact on children’s cognition and subjective experiences may play a more important role in the transition process. Such disease-specific challenges significantly affect the development of children in key areas such as self-management efficacy, health decision-making, and treatment adherence. Therefore, effective transition plans should incorporate personalized schemes based on the specific physiological, cognitive, and psychosocial needs of the disease, such as establishing a multidisciplinary collaborative intervention model that integrates mental health assessment and strong social support, to precisely enhance the effectiveness and specificity of transition interventions for different chronic disease groups.

This study found that the deterioration of disease status was in a dose-response relationship with the decline in readiness for transition, which is similar to previous research [40]. Recurrence or aggravation of the disease can lead to physical function limitations in children [41], increased stigma [42], and subsequently induce avoidance behaviors and reduce their willingness to learn and engage in self-management. At the same time, parents, out of a protective mentality, may hinder the development of disease cognition and skills in children by concealing disease information and restricting their decision-making [43]. Moreover, the high uncertainty of the future brought about by unstable disease status significantly increases the difficulty of family planning for transition [44]. These factors collectively weaken the key self-efficacy and active participation ability that children need during the transition period. Therefore, medical staff urgently need to pay attention to the psychosocial needs of children with poor disease status, especially those with aggravated conditions and their families. Therefore, this study suggests measures such as “peer support” and “mindfulness therapy” to strengthen disease management, enhance current coping abilities, help children establish rehabilitation beliefs, and improve their sense of future control, thereby stimulating their willingness and ability to actively engage in health care.

This study found that adolescents whose mothers were the primary caregivers had significantly lower transition readiness than those whose fathers were the caregivers. This finding contradicts research in Western countries [45], which may be related to cultural differences between the East and the West. In the context of traditional Chinese culture, mothers often fall into the role of “overprotection”, investing excessive care, lacking trust in their children’s abilities, and restricting their autonomy [46]. In contrast, fathers are more likely to play the role of “empowerment”, fostering their children’s independence by authorizing them to manage certain medical affairs independently (such as taking oral medication, diet, and learning about diseases). Therefore, the core strategy for optimizing transition intervention lies in precisely activating the “empowerment” role of fathers and resetting the family care roles, guiding parents to collaborate and avoid the “mother-dominated care” model, and jointly create a progressive empowerment environment for adolescents.

### Limitations

This study has the following limitations: cross-sectional studies cannot establish causal relationships between variables, and longitudinal tracking is needed to verify the dynamic association between influencing factors and transitional readiness. Secondly, the sample mainly comes from a single regional medical center in central China, which may affect the representativeness of the results and the generalizability of the conclusions. Thirdly, the data in this study are from self-reports, which may carry a certain risk of measurement bias.

## Conclusion

This study is the first to verify in the Chinese adolescent population with chronic diseases the synergistic driving effect of health care participation willingness and self-efficacy on transition readiness. The study also found that transition readiness shows a significant age gradient effect, and stratified intervention strategies need to be implemented; disease type and disease status have a key impact on the transition readiness of adolescents with chronic diseases, and psychological support should be strengthened, and individualized plans should be developed based on a multidisciplinary approach; the role of the main caregiver is significantly different, with fathers as caregivers being significantly better than mothers, suggesting the need to reshape the family collaboration mechanism. This study provides key intervention targets for the development of localized transition support plans in China. In the future, transition effectiveness can be improved through stratified empowerment, disease-customized intervention, and family role reconstruction.

## Data Availability

The data are not publicly available due to their containing information that could compromise the privacy of research participants. Data would be available upon reasonable request from the corresponding author.

## Abbreviations

STARx: The Self-Management and Transition to Adulthood with Rx-Treatment
SPSS: Statistical Package for Social Sciences
B: Unstandardized Beta
SE: Standard Error
CI: Confidence Interval
